# Knowledge, Opinion, and Practices Towards Screening of Oral Cancer Among Homeopathy and Ayurveda Students in Indore, Madhya Pradesh, India

**DOI:** 10.7759/cureus.35707

**Published:** 2023-03-02

**Authors:** Silpi Chatterjee, Asim Mustafa Khan, Reshma VJ, Muhaseena Muhamood, Muhamood Moothedath, Kumuda Rao, Faheem Muzaffar Mir

**Affiliations:** 1 Department of Public Health Dentistry, Dr. D.Y. Patil Dental College & Hospital, Dr. D.Y. Patil Vidyapeeth, Pune, IND; 2 Department of Biomedical Dental Sciences, College of Dentistry, Imam Abdulrahman Bin Faisal University, Dammam, SAU; 3 Department of Oral and Dental Health, College of Applied Health Sciences in Arrass, Qassim University, Buraidah, SAU; 4 Department of Oral Medicine and Radiology, AB Shetty Memorial Institute of Dental Sciences, Nitte University, Mangalore, IND; 5 Department of Health Policy and Health Services Research, Boston University Henry M. Goldman School of Dental Medicine, Boston, USA

**Keywords:** student, ayurveda, homeopathy, oral cancer, practices, opinion, knowledge

## Abstract

Introduction: Oral cancer is one of the most common cancers in the world. Early recognition leads to higher cure rates and better quality of life. Homeopathy and Ayurveda can help improve the general well-being and vitality of patients without inducing any side effects.

Aim: To assess knowledge, opinion, and practices towards oral cancer among homeopathy and ayurvedic students.

Objectives: To find the correlation between knowledge and practices followed by homeopathy and ayurvedic students.

Method: A descriptive cross-sectional study was conducted among 157 homeopathy and 153 ayurvedic students in Indore city, Central India. The subjects were selected using a convenient sampling technique, and the data was collected using a pre-tested close-ended self-administered questionnaire with 24 questions. The data were analyzed by IBM Corp. Released 2011. IBM SPSS Statistics for Windows, Version 20.0. Armonk, NY: IBM Corp. using descriptive and analytical (Chi-square and independent sample t-tests) statistics. Correlation analysis was performed to assess the relation between knowledge and preventive practices score.

Results: Homeopathy students showed a significantly higher mean knowledge (4.74+0.96) and practice score (4.82+1.54) as compared to ayurvedic students (knowledge score 4.49+1.11) (practice score 4.09+1.98). No significant differences were observed in the mean knowledge and practice scores of the homeopathy students. There was a statistically significant difference between the mean knowledge score and practice score of third-, fourth-, and fifth-year ayurvedic students at p-value <0.05. A positive (p-value <0.0001) correlation was observed between the knowledge and practice scores for ayurvedic students. As the year of professional studies increased, the overall practice of the students regarding oral cancer also increased, with fifth-year students showing significantly better practice (OR- 1, p-value = <0.05) than the other year students.

Conclusion: Homeopathy students showed better knowledge, whereas ayurvedic students showed better opinions and practices towards oral cancer.

## Introduction

Oral cancer is one of the most common cancers around the globe. By the end of 2020, the number of cases will be doubled, as stated by the WHO [1]. Ninety percent of oral cancers are due to tobacco usage, excessive alcohol use, and betel quid. To improve the quality of life and treatment rates, early detection and referral plays a vital role. According to the existing data, the dentist population ratio is 1:10,000 and 1:2,50,000 in urban and rural areas, respectively, which is insufficient [2]. Ayurveda is one of the most preferred branches for day-to-day healthcare needs among people of India, Nepal, and Sri Lanka. For the promotion of indigenous medicine as well as conventional biomedicine Government of India 1995 established a separate department for Indian Systems of Medicine and Homeopathy (ISM&H), which is now known as AYUSH (Ayurveda, Yoga, Unani, Siddha, Homeopathy) [3]. Since more ayurvedic and homeopathic practitioners are available in India, they can help improve the general well-being and vitality of the patient without inducing any side effects, thereby promoting an efficient way to deal with emotions as well. Also, there is a myth in society that they provide a “Magical Cure” and can treat cancer of any stage with or without metastasis by prophylactic, palliative, curative, and supportive measures, thereby enhancing the quality of life. The quality of daily practice can only be improved once the knowledge is translated into practice [4]. Various studies have been carried out among undergraduate medical and dental students [5], commerce students [1], dentists [6-8], nursing staff [9], nursing students [10], and homeopathy and ayurvedic practitioners [2]. There is scarce literature available regarding knowledge and practice among homeopathy and ayurvedic undergraduate students. Since they are future practitioners thus, the present study was carried out to assess their knowledge, opinion, and practices toward oral cancer.

## Materials and methods

A descriptive cross-sectional study was conducted among the private homeopathy and ayurveda Institutions comprising 310 subjects (157 homeopathy and 153 ayurvedic students) in Indore, Madhya Pradesh, India, from August 2015 to December 2015. Though the state includes three private homeopathy and ayurveda institutions, only two institutions offered B.H.M.S. and B.A.M.S. courses, respectively, again of which permission to conduct the study was granted by only one Institution each.

Institutional ethical board members approved the study, and ethical clearance was obtained. A convenience sampling technique was used. Two parts of the study included - designing, developing, and pilot testing the questionnaire based on construct validity, content validity, and pre-testing of the questionnaire on a group of 50 study subjects, followed by a collection of information by implementing the questionnaire. The questionnaire elicited information about age, gender, the field of study, year of study, and family history for oral cancer, if any. The questionnaire consisted of 19 closed-ended questions; six questions to assess knowledge, eight questions to assess the practices followed, and five questions to assess the opinions of the study subjects.

The questionnaire was prepared in English, containing 19 items. The questionnaires were self-administered, but a senior member of staff was present in case any subjects had any questions regarding the technical aspects of the study. The questionnaires were distributed to all of the subjects in the waiting room of the dental clinic. The response categories for all questions were “Yes” and “No”. The lowest possible score to asses knowledge for this variable was zero, and the highest possible score was six. When a respondent answered "yes" to any question, they automatically received a score of 1. A score of zero was assigned to responses of "no" or "don't know," respectively. There was a total of six questions for testing one's knowledge of oral cancer, each of which had a possible score ranging from 0 to 6, with six being the highest possible score. Similarly, for practice questions followed, the highest score assigned was eight, and zero was the lowest score noted. For questions regarding the opinion of study subjects score assigned was in a similar way. The number of questions that were answered correctly was used to determine both the group and individual scores for each question.

The overall acceptability was assessed by obtaining feedback. Based on the feedback, corrections were made to the questionnaire. The face validity of the corrected questionnaire was assessed by administering it to 50 students who were excluded from the final study. The Gutmann-split half value of 0.84 indicated good reliability of the questionnaire. The questionnaire was distributed to the students personally. The objective of the study was explained to the participants, and written informed consent was obtained. It took 5-10 minutes to complete the questionnaire. The participants present on the day of administration of the questionnaire and willing to participate in the study and providing informed written consent were included in the study.

Participants who were medically compromised and unable to respond to the questionnaire were excluded from the study. Through the use of a master file that was developed specifically to conduct data analysis, the data were transferred from precoded pro forma to a computer. Statistical analysis was done using IBM Corp. Released 2011. IBM SPSS Statistics for Windows, Version 20.0. Armonk, NY: IBM Corp. Analytical (Chi-square and independent sample t-tests) statistics were also implied. The level of significance for all the tests was set at p < 0.05. Correlation analysis was performed to assess the relationship between knowledge and preventive practices score at 0.01 level.

## Results

The study sample comprised 310 third-year, final-year, and intern students from homeopathy and ayurvedic institute. The mean age of the respondents was (in years) 23.84+1.56. The mean age for homeopathy and ayurvedic subjects were (in years) 23.84+1.56 and 23.09+2.15, respectively. Among the study subjects, 162 were females, and 148 were males. A total of 102 students from the third year, 106 subjects from the final year, and 102 students from the intern batch of both institutes participated in the study (Table 1).

**Table 1:** Distribution of the study subjects according to age, gender, year of study, and previous family history of oral cancer

Variables	Categories	Homeopathy N (%)	Ayurveda N (%)	Total	p-value
Mean age		23.84+1.56	23.09+2.15	23.84+1.56	< 0.0001
Gender	Male	77 (52.0 %)	71 (48.0 %)	148 (100.0 %)	0.642
	Female	80(49.4 %)	82 (50.6 %)	162 (100.0 %)	
Year	Third Year	52 (51.0 %)	50 (49.0 %)	102 (100.0 %)	0.987
	Fourth Year	54 (50.9 %)	52 (49.1 %)	106 (100.0 %)	
	Fifth Year	51 (50.0 %)	51 (50.0 %)	102 (100.0 %)	
Previous family history of oral cancer	Yes	2 (50.0 %)	2 (50.0 %)	4 (100.0 %)	0.979
	No	155 (50.7 %)	151 (49.3 %)	306 (100.0 %)	

About 65.6% of homeopathy students had sufficient knowledge in the prevention and detection of oral cancer as compared to ayurvedic students (32.7%). Homeopathy (98.1%) and ayurvedic (90.9%) students were aware that delayed diagnosis led to the local extension and had a risk of metastatic spread. It was seen that the percentage of subjects confident to diagnose oral cancer by clinical appearance was very low (homeopathy: 46.5% and ayurvedic: 52.3%). Most of the ayurvedic students (62%) responded that they were not adequately trained to examine patients for oral cancer. In the present study, 58.6% of homeopathy and 77.8% of ayurvedic students agreed that they were not treating oral cancer, and very few 12.1% of homeopathy and 21% of ayurvedic students had undergone training or posting in any oral cancer center (Table 2).

**Table 2:** Knowledge, opinions, and practices of the study subjects toward screening for oral cancer

Knowledge Questions	Categories	Homeopathy N (%)	Ayurveda N (%)	Total	p-value	
Is alcohol consumption a cause for oral cancer?	Yes	71 (45.2 %)	87 (56.9 %)	158 (100.0 %)		0.040
	No	86 (54.8 %)	66 (43.1 %)	152 (100.0%)		
Various forms of tobacco cause oral cancer?	Yes	156 (99.3 %)	153 (100 %)	309 (100.0 %)		0.323
	No	1 (0.7 %)	0 (0 %)	1 (100.0 %)		
Oral cancer is a disease of older age?	Yes	24 (15.2 %)	16 (10.5 %)	40 (100.0 %)		0.205
	No	133 (84.8 %)	137 (89.5 %)	270 (100.0 %)		
Early cancer clinically appears as innocuous white and/or red lesion?	Yes	128 (81.6 %)	122 (79.8 %)	250 (100.0 %)		0.690
	No	29 (18.4 %)	31 (20.2 %)	60 (100.0 %)		
Delayed diagnosis leads to local extension and has risk of metastatic spread?	Yes	154 (98.1 %)	139 (90.9 %)	293 (100.0 %)		0.005
	No	3 (1.9 %)	14 (9.1 %)	17 (100.0 %)		
Opinion Questions	Categories	Homeopathy N (%)	Ayurveda N (%)	Total		p-value
My knowledge of oral cancer is current	Yes	102 (65.0 %)	49 (32.0 %)	151 (100.0 %)		<0.0001
	No	55 (35.0 %)	104 (68.0 %)	159 (100.0 %)		
I am comfortable palpating lymph nodes in the neck	Yes	117 (74.5 %)	105 (68.6 %)	222 (100.0 %)		0.250
	No	40 (25.5 %)	48 (31.4 %)	88 (100.0 %)		
I feel confident to diagnose oral cancer by clinical appearance	Yes	84 (53.5 %)	73 (47.7 %)	157 (100.0 %)		0.308
	No	73 (46.5 %)	80 (52.3 %)	153 (100.0 %)		
I am adequately trained to provide tobacco cessation education	Yes	103 (65.6 %)	69 (45.0 %)	172 (100.0 %)		<0.0001
	No	54 (34.4 %)	84 (55.0 %)	138 (100.0 %)		
I am adequately trained to examine patients for oral cancer	Yes	71 (45.2 %)	58 (38.0 %)	129 (100.0 %)		0.191
	No	86 (54.8 %)	95 (62.0 %)	181 (100.0 %)		
Opinion Questions	Categories	Homeopathy N (%)	Ayurveda N (%)	Total		p-value
My knowledge of oral cancer is current	Yes	102 (65.0 %)	49 (32.0 %)	151 (100.0 %)		<0.0001
	No	55 (35.0 %)	104 (68.0 %)	159 (100.0 %)		
I am comfortable palpating lymph nodes in the neck	Yes	117 (74.5 %)	105 (68.6 %)	222 (100.0 %)		0.250
	No	40 (25.5 %)	48 (31.4 %)	88 (100.0 %)		
I feel confident to diagnose oral cancer by clinical appearance	Yes	84 (53.5 %)	73 (47.7 %)	157 (100.0 %)		0.308
	No	73 (46.5 %)	80 (52.3 %)	153 (100.0 %)		
I am adequately trained to provide tobacco cessation education	Yes	103 (65.6 %)	69 (45.0 %)	172 (100.0 %)		<0.0001
	No	54 (34.4 %)	84 (55.0 %)	138 (100.0 %)		
I am adequately trained to examine patients for oral cancer	Yes	71 (45.2 %)	58 (38.0 %)	129 (100.0 %)		0.191
	No	86 (54.8 %)	95 (62.0 %)	181 (100.0 %)		
Practice Questions	Categories	Homeopathy N (%)	Ayurveda N (%)	Total		p-value
Do you routinely carry out oral mucosal examination?	Yes	70 (44.6 %)	75 (49.0 %)	145 (100.0%)		0.434
	No	87 (55.4%)	78 (51.0 %)	165 (100.0%)		
Clinically have you seen any patients with oral cancer?	Yes	95 (60.5 %)	65 (42.5 %)	160 (100.0%)		0.001
	No	62 (39.5 %)	88 (57.5 %)	150 (100.0%)		
Have you undergone training or posting in any oral cancer center?	Yes	19 (12.1 %)	32 (21.0 %)	51 (100.0 %)		0.036
	No	138 (87.9 %)	121 (79.0 %)	259(100.0 %)		
Do you attend continuing educating programs/ conference regularly?	Yes	86 (54.8 %)	59 (38.6 %)	145 (100.0%)		0.004
	No	71 (45.2 %)	94 (61.4 %)	165 (100.0%)		
Do you advise patients about risk factors of oral cancer?	Yes	144 (91.7 %)	119 (77.8 %)	263 (100.0%)		0.001
	No	13 (8.3 %)	34 (22.2 %)	47 (100.0 %)		
Do you treat oral cancer?	Yes	65 (41.4 %)	34 (22.2 %)	99 (100.0 %)		<0.0001
	No	92 (58.6 %)	119 (77.8 %)	211 (100.0%)		
If you diagnosed a confirm case of oral cancer, do you refer?	Yes	127 (80.9 %)	109 (71.2 %)	236 (100.0%)		0.046
	No	30 (19.1 %)	44 (28.8 %)	74 (100.0 %)		
Would you like to receive further information / teaching on oral cancer?	Yes	150 (95.5 %)	136 (88.9 %)	286 (100.0%)		0.028
	No	7 (4.5 %)	17 (11.1 %)	24 (100.0 %)		
Do you have sufficient knowledge in prevention and detection of oral cancer?	Yes	103 (65.6 %)	50 (32.7 %)	153 (100.0 %)		<0.0001
	No	54 (34.4 %)	103 (67.3 %)	157 (100.0 %)		

No significant differences (p-value <0.05) were observed in the mean knowledge, opinion, and practice scores between the different professional years of homeopathy students. However, in Ayurvedic students, a significant difference (p-value <0.05) was observed in the mean knowledge, opinion, and practice scores between the different professional years. Post hoc analysis showed that the mean knowledge score (5.50+0.61) and mean practice score (5.49+1.61) of fifth-year ayurvedic students were significantly higher than the mean knowledge score (3.82+0.84) and mean practice score (3.48+1.96) of third-year ayurvedic students (Table 3).

**Table 3:** Comparison of knowledge and practice scores between homeopathy and ayurvedic students

Year of Study	Homeopathy		Ayurvedic	
	Knowledge Score Mean+SD	Practice Score Mean+SD	Knowledge Score Mean+SD	Practice Score Mean+SD
Third Year	4.56+0.99	4.86+1.74	3.82+0.84	3.48+1.96
Fourth Year	4.81+1.05	4.74+1.33	4.13+1.01	3.32+1.59
Fifth Year	4.84+0.83	4.84+1.55	5.50+0.61	5.49+1.61
p-Value	0.255	0.907	<0.0001	<0.0001

In ayurvedic students, a significant positive correlation was observed between knowledge-practice scores (r = 0.384, p-value = <0.0001). Also, a significant positive correlation was observed between the total knowledge and practice scores of the study subjects (r= 0.287; p-value <0.0001) (Table 4).

**Table 4:** Correlation between knowledge and practice scores of homeopathy and ayurvedic students.

Variables	Homeopathy		Ayurvedic		Total	
	Knowledge Score r (p value)	Practice Score r (p value)	Knowledge Score r (p value)	Practice Score r (p value)	Knowledge Score r (p value)	Practice Score r (p value)
Knowledge Score	-	0.109	-	0.384	-	0.287
Practice Score	0.109	-	0.384	-	0.287	-

Logistic regression analysis was performed to identify the factors that were significantly associated with good knowledge (dichotomized at >3), good opinion (dichotomized at >3), and good practice scores (dichotomized at >4). Groups and years of study were found to be significantly associated with good knowledge. Homeopathy students showed significantly better knowledge (OR: 4.16, p-value = <0.0001) than ayurvedic students. As the year of professional studies increased, the overall knowledge of the students regarding oral cancer also increased, with fifth-year students showing significantly better knowledge (OR: 1, p-value = <0.05) than the other year students. The regression model showed that the homeopathy students (OR: 1.97, p-value = 0.007) have a better opinion towards oral cancer than ayurvedic students.

It was found that groups and years of study were significantly associated with good practice. Homeopathy students showed significantly better practice (OR: 2.4, p-value = <0.0001) than ayurvedic students. As the year of professional studies increased, the overall practice of the students regarding oral cancer also increased, with fifth-year students showing significantly better practice (OR: 1, p-value = <0.05) than the other year students. No significant association was found between age and gender with knowledge, opinion, and practice scores (Table 5).

**Table 5:** Logistic regression to identify the factors significantly associated with knowledge, opinion, and practice scores

Variables		Knowledge			Opinion			Practice		
Category		Odds Ratio	CI	p-value	Odds Ratio	CI	p-value	Odds Ratio	CI	p-value
Age	<24	1.41	(0.48-4.12)	0.529	0.67	(0.35-1.28)	0.227	0.53	(0.27-1.02)	0.058
	>24	1			1			1		
Gender	Male	0.55	(0.26-1.16)	0.117	1.14	(0.69-1.89)	0.610	0.72	(0.44-1.18)	0.197
	Female	1			1			1		
Groups	Homeopath	4.16	(1.89- 9.16)*	0.000 (<0.0001)**	1.97	(1.21-3.21)	0.007*	2.4	(1.54- 3.97)	0.001
	Ayurveda	1			1			1		
Year of study	Third	0.02	(0.003-0.18)	0.001*	0.65	(0.33-1.27)	0.20	0.52	(0.27-1.02)	0.056
	Fourth	0.04	(0.005-0.37)	0.004*	0.55	(0.28-1.10)	0.09	0.45	(0.23-0.89)	0.021
	Fifth	1			1			1		

## Discussion

In the present study, fewer students (homeopathy: 44.6% and ayurveda: 49.0%) from both specialties routinely carried out an oral mucosal examination. The findings were similar to a study conducted among ayurveda and homeopathy practitioners in the Davangere district. It was seen that fewer doctors from both specialties routinely carried out an oral mucosal examination of patients who attended their practice. Thus, the oral examination should be made a compulsory part of the complete examination irrespective of the specialty [2,11]. In the present study, the percentage of homeopathy students (91.7%) advising the patients about risk factors of oral cancer was significantly higher as compared to ayurveda students (77.8%). Only 60.5% of homeopathy students and 42.5% of ayurveda students had seen patients with oral cancer clinically. This finding was also seen in the study conducted on the practitioners. Lesser number of practitioners had seen oral cancer clinically, and only 14 (33%) ayurveda and 8 (21%) homeopathy practitioners were aware of the clinical appearance of the early lesions. There was a poor understanding of the consequences of delayed diagnosis in both specialties [2,12]. Also, in the present study, homeopathy students (80.9%) were able to refer diagnosed confirmed cases of oral cancer to referral centers, whereas the percentage remained low among the ayurveda students (71.2%). This percentage was low, probably due to their lack of knowledge about the risk factors for oral cancer [13]. The students need to possess a thorough knowledge of risk factors, clinical signs, and symptoms of oral cancer to be effective in identifying, referring, and counseling high-risk patients. Oral visual screening can reduce mortality in high-risk individuals and has the potential to prevent at least 37,000 oral cancer deaths worldwide [14]. Lack of up-to-date knowledge is known to affect inconsistencies or unacceptable procedures for oral cancer examinations [15]. In the present study, only half of the study subjects were able to identify alcohol consumption as a cause of oral cancer, whereas all the students from both specialties were able to identify various forms of tobacco as a cause of oral cancer. These findings suggest strongly that educational interventions for students of both specialties are necessary [16]. The response of the students suggests the need to develop continuing education opportunities that suit the needs and wants of the study subjects [17]. The role of alcohol as a risk factor for oral cancer needs to be emphasized in future teaching of all undergraduate students [11]. It is thus apparent that further study is needed to figure out the barriers experienced by the students preventing them from imparting and implementing knowledge among the patients. Inter-professional collaboration with dental surgeons has also been suggested for both teaching and assessment [18]. The strength of this study lies in the selection of the study population, i.e., college students (youth), as they form the main source of information. This emphasizes the fact that if they are well educated about the disease, the same will also be transferred to the community at large. There is an urgent need for country-wide information, education, and communication campaign about cancer so that the general population can easily identify the initial symptoms of the disease [19].

Admission to a hospital provides an opportunity for screening for oral cancer [20]. We recommend that the students should be made well aware of the precancerous lesions so that immediate treatment can be initiated. The knowledge gained, opinions framed, and the practices followed amongst the students at different years of training can differ as curricular factors, public awareness, the role of faculty members, and society play an important role. A learning environment, including training facilities and various teaching opportunities, will enable the residents to promote the competencies and skills needed to provide an up-to-date and broad educational experience to practice in the community. In this changing era, students are expected to triage, diagnose, and treat oral diseases [10]. The limitation of the study is the lack of generalizability as it is a cross-sectional study with no ability to link findings to other factors. In the present study, overall insufficiency of knowledge, opinion, and practices followed among homeopathy and ayurveda undergraduate students was evident [12]. Thus, it is recommended that educational programs and campaigns should be organized for the betterment, enhancement, and creation of awareness among the subjects regarding oral cancer. Early diagnoses can undoubtedly make a big difference in oral cancer prevention and management.

The study limitations include the sample size and the category of the students who were only from some fraternity in a geographical area.

## Conclusions

According to the findings of this study, it was concluded that students who had studied homeopathy showed superior knowledge, whereas those who had studied ayurveda demonstrated superior opinions and practices regarding oral cancer. Students should have access to a wide variety of training facilities for the purposes of learning and promoting the competencies and skills necessary to provide an up-to-date and comprehensive educational experience that can be put into practice in the community. These facilities should be made available to students.

Because of this, there is an urgent requirement for all practitioners, regardless of their area of concentration, to significantly improve their oral cancer-related knowledge, awareness, and attitude. While students are learning about oral cancer at the undergraduate level, a greater emphasis should be placed on it. In order to raise the overall level of knowledge, it is necessary for dental schools, undergraduate and postgraduate education programs, as well as continuing education courses, to include additional guidance concerning oral cancer in all practitioners, irrespective of their specialty, in order to improve the patient's quality of life. 

## References

[1] Kulkarni GV, Angadi MM, Sorganvi VM (2013). Knowledge & attitude regarding oral cancer among college students. Int J Innov Res Dev.

[2] Kulkarni RS, Arun PD, Rai R, Kanth VS, Sargaiyan V, Kandasamy S (2015). Awareness and practice concerning oral cancer among Ayurveda and Homeopathy practitioners in Davangere District: A speciality-wise analysis. J Nat Sci Biol Med.

[3] Patwardhan K, Gehlot S, Singh G, Rathore HC (2011). The ayurveda education in India: how well are the graduates exposed to basic clinical skills?. Evid Based Complement Alternat Med.

[4] Escher M, Vollmar HC, Holling A, Raak C, Ostermann T (2012). Usage and appraisal of educational media by homeopathic therapists - a cross sectional survey. BMC Complement Altern Med.

[5] Carter LM, Ogden GR (2007). Oral cancer awareness of undergraduate medical and dental students. BMC Med Educ.

[6] Joseph BK, Sundaram DB, Sharma P (2012). Oral cancer awareness among dentists in Kuwait. Med Princ Pract.

[7] Vijay Kumar KV, Suresan V (2012). Knowledge, attitude and screening practices of general dentists concerning oral cancer in Bangalore city. Indian J Cancer.

[8] Abdullah Jaber M (2011). Dental practitioner's knowledge, opinions and methods of management of oral premalignancy and malignancy. Saudi Dent J.

[9] Carter LM, Harris AT, Kavi VP, Johnson S, Kanatas A (2009). Oral cancer awareness amongst hospital nursing staff: a pilot study. BMC Oral Health.

[10] Mittal SMA, Hiregoudar MRS, Usha M, Manjunath PG, Natraj G (2013). Knowledge of oral cancer and screening practice of B.Sc. nursing students in Davangere City, India. J Educ Ethics Dent.

[11] Mohan P (2022). UC Research Repository: Assessment of the feasibility of opportunistic screening for oral potentially malignant disorders and oral cancer at dental colleges in India : a public health initiative from Bengaluru. https://ir.canterbury.ac.nz/handle/10092/104805.

[12] Patel V, Parikh R, Nandraj S (2015). Assuring health coverage for all in India. Lancet.

[13] Tranberg M, Larsen MB, Mikkelsen EM, Svanholm H, Andersen B (2015). Impact of opportunistic testing in a systematic cervical cancer screening program: a nationwide registry study. BMC Public Health.

[14] Travasso C (2013). Betel quid chewing is responsible for half of oral cancer cases in India, finds study. BMJ.

[15] Trivedi H (1969). The “semi-urban pocket” as concept and reality in India. Hum Organ.

[16] Tsao AS, Kim ES, Hong WK (2004). Chemoprevention of cancer. CA Cancer J Clin.

[17] Turati F, Garavello W, Tramacere I (2010). A meta-analysis of alcohol drinking and oral and pharyngeal cancers. Part 2: results by subsites. Oral Oncol.

[18] Sinha DN, Gupta PC, Kumar A (2018). The poorest of poor suffer the greatest burden from smokeless tobacco use: A study from 140 countries. Nicotine Tob Res.

[19] Sinha DN, Gupta PC, Ray C, Singh PK (2012). Prevalence of smokeless tobacco use among adults in WHO South-East Asia. Indian J Cancer.

[20] Sivakumar TT, Sam N, Joseph AP (2018). Prevalence of oral potentially malignant disorders and oral malignant lesions: A population-based study in a municipal town of southern Kerala. J Oral Maxillofac Pathol.

